# Association between the triglyceride-glucose index and hyperuricemia in patients with type 2 diabetes mellitus

**DOI:** 10.3389/fendo.2025.1666563

**Published:** 2025-10-17

**Authors:** Xu Sun, Xin Li, Zhuyin Qian, Xiaowei Chen, Jie Zhang, Chenjian Zhao, Xingyu Liu

**Affiliations:** ^1^ Department of Pharmacy, Nanjing Luhe People’s Hospital, Nanjing, China; ^2^ Department of Pharmacy, Nanjing Luhe People’s Hospital, Yangzhou University, Nanjing, China; ^3^ Public Experimental Platform, China Pharmaceutical University, Nanjing, China; ^4^ Department of General Surgery, Nanjing Luhe People’s Hospital, Yangzhou University, Nanjing, China; ^5^ Department of Central Laboratory, Nanjing Luhe People’s Hospital, Yangzhou University, Nanjing, China; ^6^ Department of Endocrinology, Nanjing Luhe People’s Hospital, Yangzhou University, Nanjing, China

**Keywords:** TyG index, hyperuricemia, type 2 diabetes mellitus, statistical analysis, RCS

## Abstract

**Aim:**

This cross-sectional study investigated the relationship between the triglyceride-glucose (TyG: A surrogate marker for assessing insulin resistance.) index and hyperuricemia (HUA: Metabolic diseases caused by purine metabolism disorders.) risk in Chinese patients with type 2 diabetes mellitus (T2DM).

**Methods:**

From January 2021 to December 2023, T2DM patients were enrolled from Luhe District People’s Hospital in Nanjing. Participants were stratified by TyG index quartiles. Logistic regression and restricted cubic spline (RCS) analyses assessed the TyG-HUA association.

**Results:**

This study included 996 participants with type 2 diabetes, with a male predominance of 54.82%, a mean age of 60.39 years, and a median TyG index of 7.63. Compared to the lowest TyG quartile, the highest quartile exhibited a 4.23-fold (95% CI: 1.46 ~ 12.24, P value = 0.008) increased HUA risk. Restricted cubic spline analysis revealed a nonlinear relationship between the TyG index and HUA (nonlinear P value = 0.044). As the TyG level increased, the risk of HUA initially rose and then showed a downward trend (P for TyG = 0.008).

**Conclusions:**

Elevated TyG index independently predicts HUA risk in T2DM patients. Early metabolic intervention may mitigate HUA-related cardiovascular morbidity and mortality.

## Introduction

Hyperuricemia (HUA) is a prevalent metabolic disorder worldwide, recognized as the “fourth major metabolic abnormality” alongside hypertension, hyperglycemia, and hyperlipidemia (1, 2). Its comorbidity with type 2 diabetes mellitus (T2DM) is particularly pronounced, with a 19% HUA prevalence among T2DM patients, driven by shared pathophysiological mechanisms including insulin resistance (IR), obesity, and dyslipidemia (3–5).

The triglyceride-glucose (TyG) index, calculated as ln[fasting triglycerides (TG, mg/dL) × fasting blood glucose (FBG, mg/dL)/2], is a validated surrogate marker of IR and systemic metabolic dysregulation (6, 7). In T2DM populations, elevated TyG index not only predicts diabetic nephropathy risk but also correlates with aberrant uric acid metabolism (8, 9). Mechanistically (10, 11), TyG-reflected IR promotes hepatic purine metabolism (increasing uric acid production) and inhibits renal tubular uric acid excretion, leading to serum uric acid accumulation (12–14). Clinical evidence confirms a strong positive correlation between TyG index and serum uric acid levels (15, 16).For example: Luo Y et al. found a positive correlation between the TyG index and SUA levels in non-obese individuals with type 2 diabetes. Additionally, TyG may outperform HOMA-IR in predicting HUA in this population (17). Among patients with non-alcoholic fatty liver disease (NAFLD), every 0.1-unit increase in TyG corresponded to a 1.53-unit elevation in SUA levels. Further, the TyG index was identified as an independent risk factor for HUA development in NAFLD patients. As a readily available metric, TyG can help identify high-risk individuals for NAFLD progression, potentially reducing the incidence of HUA and related complications (18). In a study of a physical examination cohort in Xinjiang, China, TyG index demonstrated stronger correlation with HUA than nine obesity indices and showed superior performance over these indices in detecting HUA (19).

Thus, the TyG-HUA association in T2DM epitomizes the insulin resistance–lipotoxicity–end-organ damage cascade. This study aims to elucidate this relationship in a Chinese T2DM cohort to facilitate early risk stratification and integrated metabolic comorbidity management.

## Materials and methods

### Study participants

**Figure 1:**
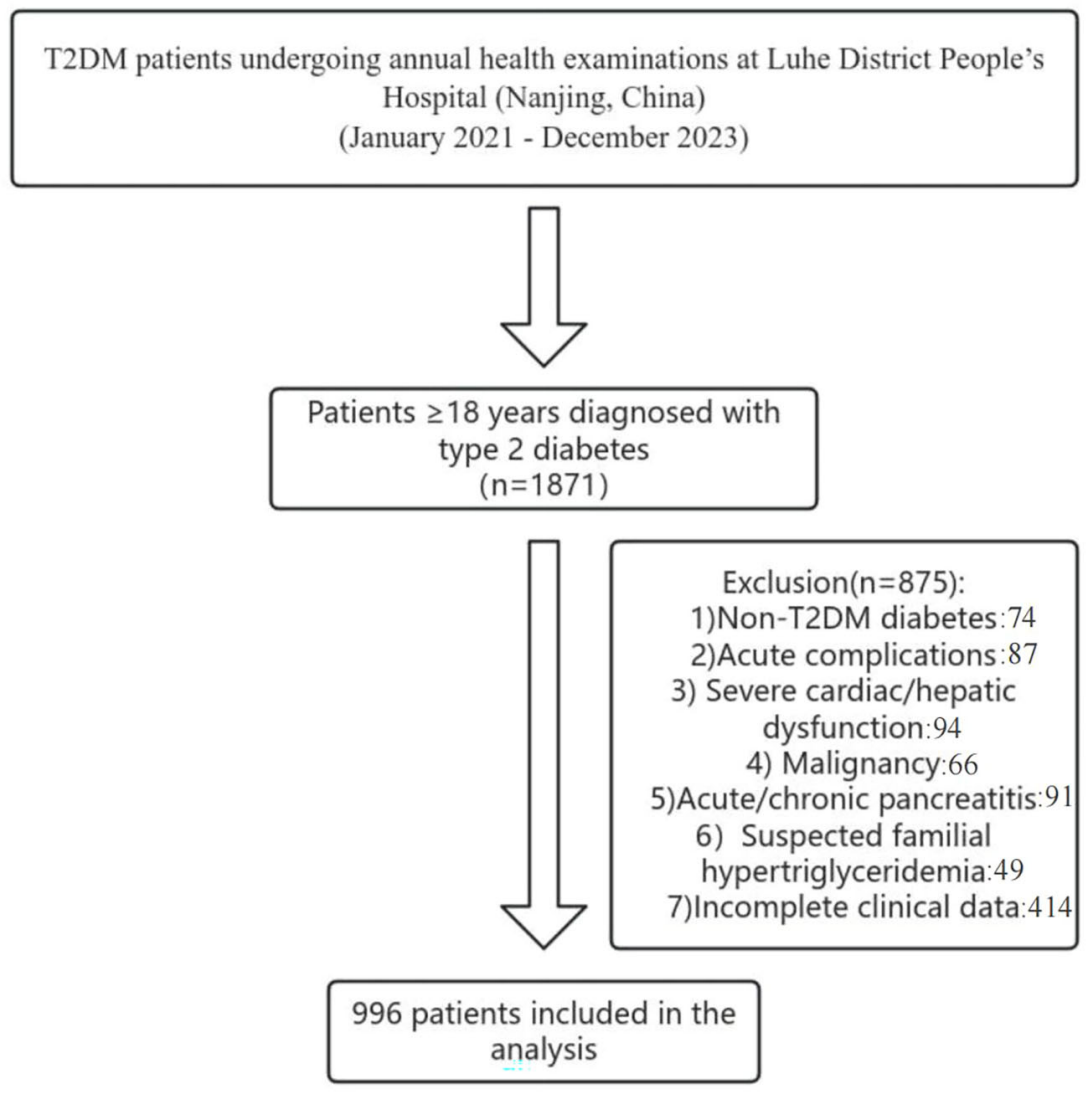
Patient screening flowchart.

We conducted a cross-sectional study of T2DM patients undergoing annual health examinations at Liuhe District People’s Hospital (Nanjing, China) between January 2021 and December 2023. Inclusion criteria: Adults ≥18 years meeting WHO T2DM diagnostic criteria or previously diagnosed T2DM (20). Exclusion criteria: Non-T2DM diabetes; acute complications (e.g., ketoacidosis, hyperosmolar coma); severe cardiac/hepatic dysfunction(Severe cardiac dysfunction refers to a state of severely reduced cardiac pumping capacity, leading to inadequate systemic organ perfusion and a substantially increased risk of multi-organ failure;Severe hepatic dysfunction denotes a critical loss of the liver’s synthetic, metabolic, and detoxification functions, often accompanied by portal hypertension and high-risk stratification in end-stage liver disease scoring systems); malignancy; acute/chronic pancreatitis; suspected familial hypertriglyceridemia; incomplete clinical data. From 1,871 initially screened, 996 patients were included(As shown in the following **Figure 1**). All eligible patients were consecutively screened and enrolled.

Ethical approval and consent to participate.

The research protocol was developed in accordance with the relevant requirements of the World Medical Association’s Declaration of Helsinki. The research protocol was reviewed and approved by the Ethics Committee of Nanjing Liuhe District People’s Hospital, Jiangsu Province (Ethics Number: LHLL0029), and all participants signed the informed consent form. This process ensures compliance with ethical regulations (**Supplementary Figure S1**).

### Data definitions

TyG Index (21): In[TG (mg/dL) × FBG (mg/dL)/2] (conversion: TG: 1 mmol/L = 88.57 mg/dL; FBG: 1 mmol/L = 18 mg/dL).

T2DM (22): (1) Classic symptoms + random glucose ≥11.1 mmol/L or FBG ≥7.0 mmol/L or 2-h OGTT ≥11.1 mmol/L; (2) Asymptomatic patients required confirmatory testing.

HUA (23): the diagnosis of hyperuricemia (HUA) strictly follows the recommended criteria in the “Multidisciplinary Expert Consensus on Diagnosis and Treatment of Hyperuricemia-related Diseases (2023 Edition)” in China, which is: in a normal purine diet state, fasting blood uric acid levels > 420 μmol/L on two separate days. This standard is gender-neutral and uniformly applicable to all adult patients. Regarding the time interval of “not on the same day”, we follow clinical practice and define it as two independent tests at least one week apart.

25-hydroxyvitamin D standard: serum 25(OH)D levels (24): sufficient (≥30 ng/mL [75 nmol/L]), insufficient (20–29 ng/mL [50–74 nmol/L]), and deficient (<20 ng/mL [50 nmol/L]).Covariates:The covariates that may influence the association between the TyG index and kidney disease include age (Age, years), gender (Sex, male/female), hypertension (Hypertension, yes/no), body mass index (BMI, kg/m^2^), systolic blood pressure (SBP, mmHg), diastolic blood pressure (DBP, mmHg), hemoglobin A1c (HbA1c,%), total cholesterol (TC, mmol/L), triglycerides (TG, mmol/L), high-density lipoprotein cholesterol (HDL-C, mmol/L), low-density lipoprotein cholesterol (LDL-C, mmol/L), lipoprotein a (Lp(a), mg/dL), alanine aminotransferase (ALT, IU/L), aspartate aminotransferase (AST, IU/L), fasting blood glucose (FBG, mmol/L), urea (UREA, mmol/L), serum creatinine (Scr, mmol/L), serum uric acid (SUA, μmol/L), glomerular filtration rate (eGFR,mL/min/1.73m^2^), urine albumin creatinine ratio (UACR, mg/g), blood calcium (Ca, mmol/L), blood phosphorus (P, mmol/L), parathyroid hormone (PTH, ng/L), and 25-hydroxyvitamin D (25(OH)D, ng/mL).

### Statistical analysis

Data were analyzed using SPSS 23.0 and R 4.2.2. Continuous variables are expressed as mean ± SD or median (IQR); categorical variables as frequencies (%). Group differences were assessed via ANOVA, Kruskal-Wallis, or χ² tests (25). Multivariable logistic regression estimated OR and 95% CI for TyG-HUA associations across three models:Model 1: Adjusted for age, sex;Model 2: Model 1 + BMI, SBP, DBP, HbA1c, lipids, liver enzymes, renal markers, minerals, 25(OH)D;Model 3: Model 2 + eGFR.Multicollinearity was excluded (VIF < 5). Restricted cubic splines (RCS) evaluated nonlinearity. Subgroup (sex, hypertension) and sensitivity (vitamin D deficiency) analyses were performed. Significance: two-tailed P < 0.05.Furthermore, subgroup analysis and sensitivity analysis were conducted among individuals lacking vitamin D to further confirm the aforementioned relationship. In summary, this section provides a detailed account of the statistical methods employed in the study, from the description and comparison of variables to the construction of regression models and the assessment of multicollinearity. It offers a comprehensive and systematic exploration of the relationship between the TyG index and HUA, providing robust statistical support for the research conclusions.

## Results

### Baseline characteristics

The cohort (*n* = 996) had a mean age of 60.39 ± 13.34 years; 54.82% were male. Higher TyG quartiles were associated with younger age, elevated BMI, blood pressure, HbA1c, LDL-C, liver enzymes, FBG, uric acid, eGFR, and calcium, but lower HDL-C and 25(OH)D (P < 0.05; **Table 1**).

**Table 1:** Baseline characteristics of the quartiles of the TyG index.

Variables	Total (n = 996)	1 (n = 249)	2 (n = 249)	3 (n = 249)	4 (n = 249)	*P*
Age(years), Mean ± SD	60.39 ± 13.34	63.96 ± 11.22	62.19 ± 12.02	60.24 ± 12.80	55.17 ± 15.36	<.001
Sex, n(%)						0.176
Male	546 (54.82)	150 (60.24)	131 (52.61)	127 (51.00)	138 (55.42)	
Female	450 (45.18)	99 (39.76)	118 (47.39)	122 (49.00)	111 (44.58)	
Hypertension, n(%)						0.508
No	493 (49.50)	117 (46.99)	133 (53.41)	120 (48.19)	123 (49.40)	
Yes	503 (50.50)	132 (53.01)	116 (46.59)	129 (51.81)	126 (50.60)	
BMI(kg/m^2^), Mean ± SD	24.64 ± 3.94	23.52 ± 3.68	24.51 ± 3.80	25.02 ± 4.15	25.51 ± 3.85	<.001
SBP(mmHg), Mean ± SD	136.91 ± 18.36	136.06 ± 18.52	136.34 ± 17.88	136.00 ± 18.73	139.25 ± 18.18	0.142
DBP(mmHg), Mean ± SD	82.35 ± 13.35	79.88 ± 12.19	81.15 ± 12.53	82.58 ± 13.35	85.77 ± 14.56	<.001
HbA1c(%), Mean ± SD	10.04 ± 2.53	9.39 ± 2.78	9.68 ± 2.52	10.21 ± 2.38	10.86 ± 2.18	<.001
TC(mmol/L), Mean ± SD	4.94 ± 3.64	4.11 ± 1.09	4.46 ± 1.14	5.23 ± 5.47	5.95 ± 4.32	<.001
TG(mmol/L), Mean ± SD	2.03 ± 2.49	0.85 ± 0.32	1.29 ± 0.39	1.76 ± 0.63	4.24 ± 4.16	<.001
HDL-C(mmol/L), Mean ± SD	1.14 ± 0.33	1.29 ± 0.37	1.16 ± 0.30	1.10 ± 0.25	1.02 ± 0.31	<.001
LDL-C(mmol/L), Mean ± SD	2.89 ± 1.03	2.46 ± 0.91	2.83 ± 0.92	3.06 ± 0.97	3.21 ± 1.14	<.001
Lp(a)(mmol/L), Mean ± SD	218.20 ± 258.61	240.13 ± 268.31	253.54 ± 291.88	229.77 ± 262.37	149.35 ± 188.88	<.001
ALT(IU/L), Mean ± SD	25.44 ± 24.83	20.28 ± 14.01	24.66 ± 24.12	25.42 ± 21.70	31.39 ± 34.08	<.001
AST(IU/L), Mean ± SD	23.47 ± 18.40	20.92 ± 9.34	23.52 ± 17.73	23.29 ± 20.87	26.16 ± 22.52	0.017
FBG(mmol/L), Mean ± SD	9.48 ± 4.36	5.50 ± 2.00	8.24 ± 2.80	10.98 ± 3.72	13.21 ± 4.14	<.001
UREA(mmol/L), Mean ± SD	7.42 ± 16.18	6.31 ± 2.64	7.18 ± 14.12	8.30 ± 21.83	7.87 ± 19.11	0.541
Scr(mmol/L), Mean ± SD	73.63 ± 33.91	75.07 ± 38.90	73.83 ± 33.20	71.17 ± 26.25	74.46 ± 36.04	0.59
SUA(μmol/L), Mean ± SD	293.85 ± 100.09	271.41 ± 88.99	282.79 ± 99.41	298.26 ± 103.71	322.95 ± 100.69	<.001
eGFR(mL/min/1.73m^2^), Mean ± SD	96.76 ± 50.29	88.58 ± 61.19	90.66 ± 40.59	97.44 ± 39.31	110.34 ± 54.04	<.001
UACR(mg/g), Mean ± SD	49.00 ± 298.27	61.12 ± 416.30	54.78 ± 371.13	34.94 ± 143.82	45.14 ± 157.53	0.778
Ca(mmol/L), Mean ± SD	2.29 ± 0.14	2.24 ± 0.13	2.28 ± 0.13	2.31 ± 0.14	2.33 ± 0.15	<.001
P(mmol/L), Mean ± SD	1.12 ± 0.22	1.13 ± 0.20	1.13 ± 0.21	1.12 ± 0.23	1.10 ± 0.22	0.256
PTH(ng/L), Mean ± SD	45.60 ± 21.51	46.62 ± 24.77	44.36 ± 18.78	46.55 ± 21.88	44.85 ± 20.17	0.535
25(OH)D(ng/mL), Mean ± SD	20.02 ± 6.08	20.79 ± 6.80	20.59 ± 6.01	19.55 ± 5.70	19.16 ± 5.61	0.005

### Collinearity assessment and logistic regression results

Collinearity diagnostics revealed no significant multicollinearity among covariates, with all variance inflation factors (VIF) < 5 (**Supplementary Table S1**).

Logistic regression results are presented in **Table 2**. Logistic regression analyses(Crude model) demonstrated a consistent positive association between the TyG index and HUA: Each unit increase in TyG index was associated with a 33% elevated risk of HUA (OR = 1.33, 95% CI: (1.10 ~ 1.61)). Adjusted models:Model 1 (adjusted for age and sex): Higher TyG index significantly increased HUA risk (OR = 2.85, 95% CI: 1.57–5.19; P < 0.001).Model 2 (Model 1 + BMI, SBP, DBP, HbA1c, lipids, liver enzymes, renal markers, minerals, 25(OH)D): Participants in the highest TyG quartile had a 4.45-fold higher HUA risk versus the lowest quartile (OR = 4.45, 95% CI: 1.54–12.86; P = 0.006).Model 3 (Model 2 + eGFR): The association persisted (Q4 vs. Q1: OR = 4.23, 95% CI: 1.46–12.24; P = 0.008).Key conclusion: The TyG index remained an independent predictor of HUA risk after multivariable adjustment, with progressively increasing risk across ascending TyG quartiles.

**Table 2:** Association between TyG and HUA.

Variables	Unadjusted model		Model 1		Model 2		Model 3	
	OR (95%CI)	*P*	OR (95%CI)	*P*	OR (95%CI)	*P*	OR (95%CI)	*P*
TyG index	1.33 (1.10 ~ 1.61)	0.003	1.39 (1.14 ~ 1.70)	0.001	1.92 (1.11 ~ 3.30)	0.019	1.88 (1.09 ~ 3.26)	0.024
TyG index quantile								
Q1	1.00 (Reference)		1.00 (Reference)		1.00 (Reference)		1.00 (Reference)	
Q2	1.50 (0.80 ~ 2.81)	0.209	1.43 (0.75 ~ 2.69)	0.275	1.62 (0.71 ~ 3.71)	0.25	1.55 (0.69 ~ 3.52)	0.29
Q3	2.24 (1.24 ~ 4.05)	0.008	2.16 (1.18 ~ 3.95)	0.012	3.24 (1.38 ~ 7.62)	0.007	3.06 (1.31 ~ 7.18)	0.01
Q4	2.68 (1.50 ~ 4.79)	<.001	2.85 (1.57 ~ 5.19)	<.001	4.45 (1.54 ~ 12.86)	0.006	4.23 (1.46 ~ 12.24)	0.008
P for trend	1.38 (1.16 ~ 1.65)	<.001	1.42 (1.19 ~ 1.71)	<.001	1.69 (1.21 ~ 2.36)	0.002	1.66 (1.19 ~ 2.33)	0.003

Model 1: Adjust: Age, Sex.

Model 2: Adjust: Age, Sex, BMI, SBP, DBP, HbA1c, TC, TG, HDL.C, LDL.C, Lp.a, ALT, AST, FBG, UREA, Scr, UACR, Ca, P, PTH,25(OH)D.

Model3: Adjust: Age, Sex, BMI, SBP, DBP, HbA1c, TC, TG, HDL.C, LDL.C, Lp.a, ALT, AST, FBG, UREA, Scr, UACR, Ca, P, PTH,25(OH)D, eGFR.

**Figure 2:**
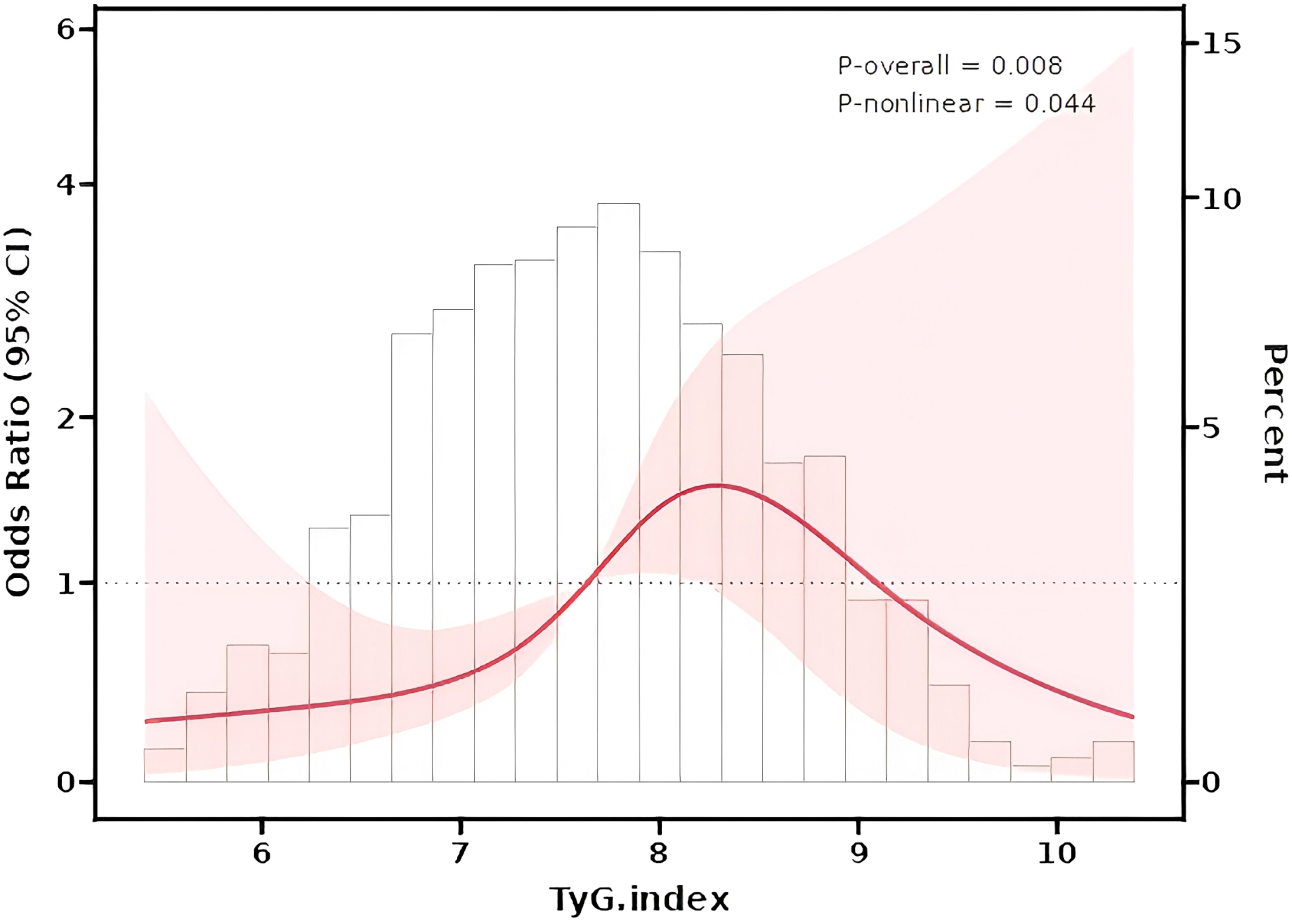
Restrcited cubic splines (RCS) for the shape of the association of TyG index and HUA risk.

Nonlinear Relationship Analysis: Restricted cubic splines (RCS) confirmed a nonlinear relationship between the TyG index and HUA risk (The nonlinear P value was 0.044; **Figure 2**). The number of knots used in the RCS analysis is 4, and the inflection point value is 7.6.The dose-response curve exhibited an inverted U-shape: HUA risk initially rose with increasing TyG levels but declined at higher values (The P value of TyG was 0.008).

### Subgroup and sensitivity analyses

**Figure 3:**
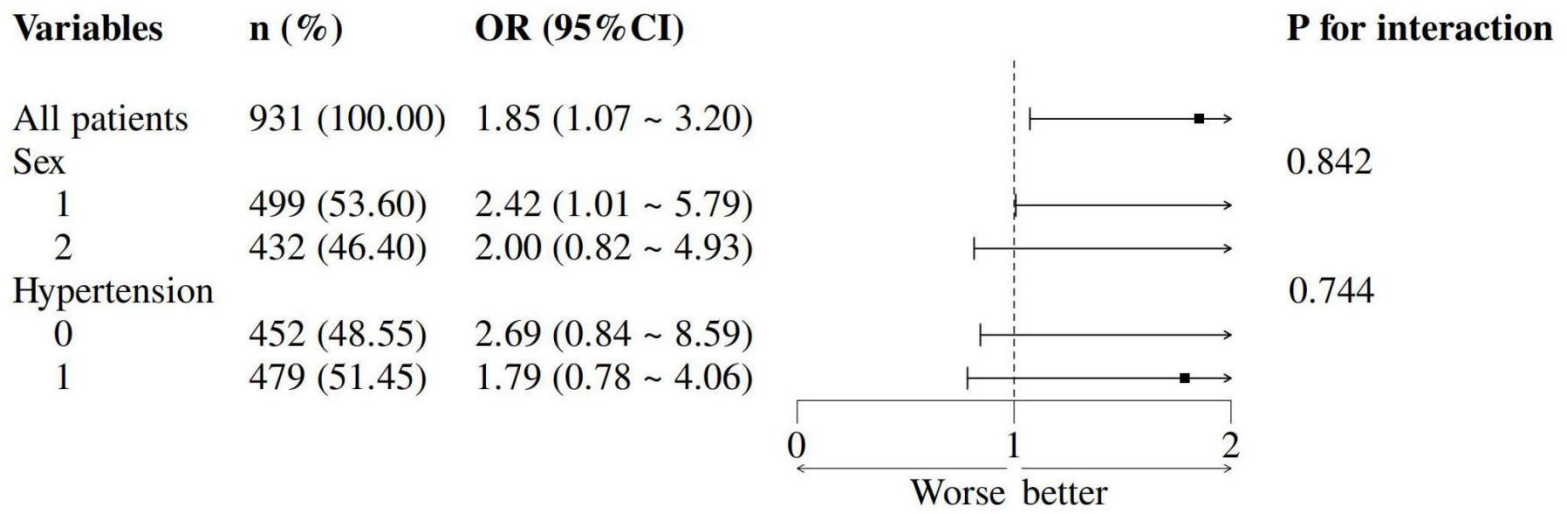
Subgroup analysis of the correlation between TyG index and HUA.

The TyG-HUA association remained consistent across sex and hypertension subgroups (all P_interaction_ > 0.05; **Figure 3**). Sensitivity analysis in vitamin D-deficient patients yielded stable results (**Supplementary Table S2**).

## Discussion

This study establishes an independent and nonlinear association between elevated TyG index and HUA risk in T2DM patients, persisting after rigorous adjustment for confounders and across analytical methods (quartile/continuous TyG).

Additionally, the relationship between the TyG index and the risk of HUA is nonlinear, where HUA first increases and then decreases as the TyG index rises. This phenomenon primarily stems from the transition of metabolic compensation mechanisms and stage-specific organ dysfunction. Early Stage: HUA Increases with Rising TyG Index. Increased uric acid production (26, 27): A higher TyG index indicates insulin resistance and glycolipid metabolic disorders, leading to increased free fatty acid release. This enhances hepatic triglyceride synthesis and purine metabolism, elevating uric acid production (10). Reduced uric acid excretion: Insulin resistance inhibits renal uric acid excretion, while hyperinsulinemia promotes renal reabsorption of sodium and uric acid, further raising serum uric acid levels (28–30). The TyG index often correlates with visceral obesity (increased waist circumference). Adipose tissue releases inflammatory cytokines and free fatty acids (31), which not only worsen insulin resistance but also enhance uric acid production by activating purine metabolic pathways. Hypertriglyceridemia causes accumulation of ketone bodies and lactate, competitively inhibiting uric acid excretion in renal tubules while promoting hepatic uric acid synthase activity (32). Late Stage: HUA decreases with sustained high TyG Index. Prolonged HUA induces urate crystal deposition in renal tubules, leading to chronic kidney disease (33). As glomerular filtration rate declines, total uric acid excretion decreases. Advanced metabolic syndrome patients often develop complications like diabetic nephropathy and cardiac insufficiency, with significantly reduced systemic metabolic function. This weakens hepatic uric acid synthesis capacity (34). Although the TyG index remains high, uric acid production decreases due to overall metabolic failure.

The experimental findings of this study align with the majority of relevant literature regarding the positive correlation between the TyG index and serum uric acid levels. However, our research incorporated a wider range of covariates in analyzing the TyG-uric acid relationship, thereby enhancing the accuracy of risk prediction models and significantly strengthening the scientific rigor and clinical relevance of the study. Notably, this investigation has limitations. The study sample was exclusively drawn from Nanjing, introducing geographical constraints. Nevertheless, given the relatively homogeneous nature of the study population, the conclusions should maintain validity.

The results of this cross-sectional study provide an important hypothesis for future prospective cohort studies and randomized controlled trials: whether reducing the TyG index through lifestyle changes can effectively prevent or delay the occurrence of hyperuricemia in T2DM patients. If this hypothesis is confirmed, the TyG index is expected to become a practical biomarker for HUA risk stratification and intervention effect evaluation.

## Conclusions

Higher TyG index independently predicts HUA risk in type 2 diabetics, exhibiting a nonlinear trajectory. Integrating TyG assessment into T2DM management may optimize metabolic control and reduce HUA-related complications. Further studies should validate its clinical utility in diverse populations.

## Data Availability

The original contributions presented in the study are included in the article/**Supplementary Material**. Further inquiries can be directed to the corresponding author.

## Supplementary material

Supplementary Figure 1 Ethical approval and consent to participate.

## References

[1] Li L Zhang Y Zeng C. Update on the epidemiology, genetics, and therapeutic options of hyperuricemia. Am J Transl Res. (2020) 12:3167–81., PMID: 32774692 PMC7407685

[2] Zhou H Yang J Yuan X Song X Zhang X Cao T. Hyperuricemia research progress in model construction and traditional Chinese medicine interventions. Front Pharmacol. (2024) 15:1294755. doi: 10.3389/fphar.2024.1294755, PMID: 38515855 PMC10955118

[3] Li C Hsieh MC Chang SJ. Metabolic syndrome, diabetes, and hyperuricemia. Curr Opin Rheumatol. (2013) 25:210–6. doi: 10.1097/BOR.0b013e32835d951e, PMID: 23370374

[4] Yang H Ying J Zu T Meng XM Jin J. Insights into renal damage in hyperuricemia: Focus on renal protection (Review). Mol Med Rep. (2025) 31:59. doi: 10.3892/mmr.2024.13424, PMID: 39717954 PMC11711934

[5] Choi HK McCormick N Yokose C. Excess comorbidities in gout: the causal paradigm and pleiotropic approaches to care. Nat Rev Rheumatol. (2022) 18:97–111. doi: 10.1038/s41584-021-00725-9, PMID: 34921301

[6] Li C Zhang Z Luo X Xiao Y Tu T Liu C. The triglyceride-glucose index and its obesity-related derivatives as predictors of all-cause and cardiovascular mortality in hypertensive patients: insights from NHANES data with machine learning analysis. Cardiovasc Diabetol. (2025) 24:47. doi: 10.1186/s12933-025-02591-1, PMID: 39881352 PMC11780913

[7] Li HF Miao X Li Y. The triglyceride glucose (TyG) index as a sensible marker for identifying insulin resistance and predicting diabetic kidney disease. Med Sci Monit. (2023) 29:e939482. doi: 10.12659/MSM.939482, PMID: 37421131 PMC10337482

[8] Shi W Xing L Jing L Tian Y Liu S. Usefulness of Triglyceride-glucose Index for estimating Hyperuricemia risk: Insights from a general Population. Postgrad Med. (2019) 131:348–56. doi: 10.1080/00325481.2019.1624581, PMID: 31132018

[9] Gou R Dou D Tian M Chang X Zhao Y Meng X. Association between triglyceride glucose index and hyperuricemia: a new evidence from China and the United States. Front Endocrinol (Lausanne). (2024) 15:1403858. doi: 10.3389/fendo.2024.1403858, PMID: 39010899 PMC11246899

[10] Li Q Shao X Zhou S Cui Z Liu H Wang T. Triglyceride-glucose index is significantly associated with the risk of hyperuricemia in patients with diabetic kidney disease. Sci Rep. (2022) 12:19988. doi: 10.1038/s41598-022-23478-1, PMID: 36411302 PMC9678876

[11] Zhang R Peng J Wu Q Zhu H Zhang Z Feng Y. Association between the combination of the triglyceride-glucose index and obesity-related indices with hyperuricemia among children and adolescents in China. Lipids Health Dis. (2025) 24:150. doi: 10.1186/s12944-025-02547-0, PMID: 40269945 PMC12016125

[12] Zhang C Li L Zhang Y Zeng C. Recent advances in fructose intake and risk of hyperuricemia. BioMed Pharmacother. (2020) 131:110795. doi: 10.1016/j.biopha.2020.110795, PMID: 33152951

[13] Li X Wang L Zhou H Xu H. Association between triglyceride-glucose index and chronic kidney disease: results from NHANES 1999-2020. Int Urol Nephrol. (2024) 56:3605–16. doi: 10.1007/s11255-024-04103-8, PMID: 38856937 PMC11464617

[14] Dong J Yang H Zhang Y Hu Q. Triglyceride-glucose index is a predictive index of hyperuricemia events in elderly patients with hypertension: a cross-sectional study. Clin Exp Hypertens. (2022) 44:34–9. doi: 10.1080/10641963.2021.1984499, PMID: 34633263

[15] Lertsakulbunlue S Sangkool T Bhuriveth V Mungthin M Rangsin R Kantiwong A. Associations of triglyceride-glucose index with hyperuricemia among Royal Thai Army personnel. BMC Endocr Disord. (2024) 24:17. doi: 10.1186/s12902-024-01542-3, PMID: 38297286 PMC10832246

[16] Wang J He Q Sun W Li W Yang Y Cui W. The association between the triglyceride glucose index and hyperuricemia: A dose-response meta-analysis. Nutrients. (2025) 17:1462. doi: 10.3390/nu17091462, PMID: 40362772 PMC12073563

[17] Luo Y Hao J He X. Association between triglyceride-glucose index and serum uric acid levels: a biochemical study on anthropometry in non-obese type 2 diabetes mellitus patients. DMSO. (2022) 15:3447–58. doi: 10.2147/DMSO.S387961, PMID: 36353666 PMC9639381

[18] Qi J Ren X Hou Y Zhang Y Zhang Y Tan E. Triglyceride-glucose index is significantly associated with the risk of hyperuricemia in patients with nonalcoholic fatty liver disease. Diabetes Metab Syndr Obes. (2023) 16:1323–34. doi: 10.2147/DMSO.S408075, PMID: 37188227 PMC10179341

[19] Kahaer M Zhang B Chen W Liang M He Y Chen M. Triglyceride glucose index is more closely related to hyperuricemia than obesity indices in the medical checkup population in Xinjiang, China. Front Endocrinol. (2022) 2:861760. doi: 10.3389/fendo.2022.861760, PMID: 35311243 PMC8924289

[20] Xu L Ran J Shao H Chen M Tang H Li Y. Incidence and risk factors of diagnosed young-adult-onset type 2 diabetes in the U.S.: the national health interview survey 2016-2022. Diabetes Care. (2025) 48:371–80. doi: 10.2337/dc24-1699, PMID: 39752552 PMC12328765

[21] Liu D Ren B Tian Y Chang Z Zou T. Association of the TyG index with prognosis in surgical intensive care patients: data from the MIMIC-IV. Cardiovasc Diabetol. (2024) 23:193. doi: 10.1186/s12933-024-02293-0, PMID: 38844938 PMC11157750

[22] American Diabetes Association. Classification and diagnosis of diabetes: standards of medical care in diabetes-2021. Diabetes Care. (2021) 44:S15–33. doi: 10.2337/dc21-S002, PMID: 33298413

[23] Kjaergaard AD Smith GD Stewart P. Mendelian randomization studies in endocrinology: raising the quality bar for submissions and publications in the journal of clinical endocrinology & Metabolism. J Clin Endocrinol Metab. (2023) 109:1–3. doi: 10.1210/clinem/dgad569, PMID: 37796951

[24] Wang R Xu F Xia X Xiong A Dai D Ling Y. The effect of vitamin D supplementation on primary depression: A meta-analysis. J Affect Disord. (2024) 344:653–61. doi: 10.1016/j.jad.2023.10.021, PMID: 37852593

[25] Li M Lian B Xu X Zhao P Tang B Hu C. Collaborative relationships in translational medical research among Chinese clinicians: an internet-based cross-sectional survey. J Transl Med. (2021) 19:247. doi: 10.1186/s12967-021-02911-5, PMID: 34090449 PMC8180016

[26] Yanai H Adachi H Hakoshima M Katsuyama H. Molecular biological and clinical understanding of the pathophysiology and treatments of hyperuricemia and its association with metabolic syndrome, cardiovascular diseases and chronic kidney disease. Int J Mol Sci. (2021) 22:9221. doi: 10.3390/ijms22179221, PMID: 34502127 PMC8431537

[27] Washio KW Kusunoki Y Murase T Nakamura T Osugi K Ohigashi M. Xanthine oxidoreductase activity is correlated with insulin resistance and subclinical inflammation in young humans. Metabolism. (2017) 70:51–6. doi: 10.1016/j.metabol.2017.01.031, PMID: 28403945

[28] Ponticelli C Podestà MA Moroni G. Hyperuricemia as a trigger of immune response in hypertension and chronic kidney disease. Kidney Int. (2020) 98:1149–59. doi: 10.1016/j.kint.2020.05.056, PMID: 32650020

[29] Kang DH Nakagawa T Feng L Watanabe S Han L Mazzali M. A role for uric acid in the progression of renal disease. J Am Soc Nephrol. (2002) 13:2888–97. doi: 10.1097/01.asn.0000034910.58454.fd, PMID: 12444207

[30] Vidal-Ostos F Ramos-Lopez O Blaak EE Astrup A Martinez JA. The triglyceride-glucose index as an adiposity marker and a predictor of fat loss induced by a low-calorie diet. Eur J Clin Invest. (2022) 52:e13674. doi: 10.1111/eci.13674, PMID: 34453322

[31] Yu P Yuan H Li X Chen H. Impact of cortisol on liver fat and metabolic health in adrenal incidentalomas and Cushing’s syndrome. Endocrine. (2025) 87:334–43. doi: 10.1007/s12020-024-04043-4, PMID: 39320593

[32] Mei Y Dong B Geng Z Xu L. Excess uric acid induces gouty nephropathy through crystal formation: A review of recent insights. Front Endocrinol (Lausanne). (2022) 13:911968. doi: 10.3389/fendo.2022.911968, PMID: 35909538 PMC9329685

[33] Cui D Liu S Tang M Lu Y Zhao M Mao R. Phloretin ameliorates hyperuricemia-induced chronic renal dysfunction through inhibiting NLRP3 inflammasome and uric acid reabsorption. Phytomedicine. (2020) 66:153111. doi: 10.1016/j.phymed.2019.153111, PMID: 31790902

[34] Silveira Rossi JL Barbalho SM Reverete de Araujo R Bechara MD Sloan KP Sloan LA. Metabolic syndrome and cardiovascular diseases: Going beyond traditional risk factors. Diabetes Metab Res Rev. (2022) 38:e3502. doi: 10.1002/dmrr.3502, PMID: 34614543

