# Supplementary material for: Association between the triglyceride-glucose index and hyperuricemia in patients with type 2 diabetes mellitus
Source: Front Endocrinol (Lausanne). 2025 Oct 17;16:1666563. doi: 10.3389/fendo.2025.1666563 (PMC12575192; doi:10.3389/fendo.2025.1666563)
Supplement: Supplementary Figure 1 — Ethical approval and consent to participate. [file Image1.pdf]

Nanjing Luhe District People's Hospital

Medical Ethics Committee Review and Approval Form

No.:(LHLL2021029)

Project name: Association between the triglyceride-glucose index and hyperuricemia in patients with type 2 diabetes mellitus

Key researchers and institutions: Xu Sun, Nanjing Luhe District People's Hospital

1. Elements of Informed Consent:

	**The basic elements of informed consent**	have	not have
1	The experiment is of a research nature	√	
2	purpose of research	√	
3	The possibility of trial treatment and randomization into groups	√	
4	The procedure of the experiment, including all traumatic operations	√	
5	Subject responsibilities	√	
6	Description of the trial intervention/procedural	√	
7	Reasonable anticipated risk or inconvenience to the subject (including to embryos, fetuses or breastfed infants, if necessary)	√	
8	Reasonable anticipated benefit. If no anticipated benefit is expected for the subject, this should be made known	√	
9	Other alternative treatments or therapies that the subject may receive, and their important benefits and risks	√	
10	Compensation and/or treatment that may be available to the subject in the event of an injury related to the trial	√	
11	Remuneration (if any) to be paid proportionately to the subject for participation in the study	√	
12	The expected cost to the subject to participate in the trial (if any)	√	
13	Participants are voluntary and may refuse to participate or withdraw from the trial at any time without penalty or loss of their entitlements	√	
14	Investigators, auditors, institutional review boards/independent ethics committees, and management authorities shall be permitted to access participants 'original medical records directly within the scope permitted by applicable laws and regulations, provided that such access does not violate participants' privacy. This authorization for record review is granted when participants or their legally authorized representatives sign written informed consent forms.	√	
15	Within the scope permitted by applicable laws and/or regulations, records identifying subjects shall be kept confidential and shall not be made public, such as publication of trial results, and the identity of subjects shall remain confidential	√	
16	If information is obtained that may affect the subject's continued participation in the trial, the subject or his/her legal representative will be notified in a timely manner	√	
17	Need to further understand the contact person for information about the trial and the rights and interests of the subject, as well as the contact person in case of trial-related injury	√	
18	The expected circumstances and/or reasons for the termination of a subject's participation in the trial	√	

19	The expected duration of the subject's participation in the trial	√	
20	The study involves the approximate number of subjects	√	

2. Other Questions About Informed Consent:

(1) Is the informed consent form adequate?

☑yes ☐no

(2) Is the language and presentation of the information being communicated appropriate for the subject group (considering literacy, use of complex sentence patterns and technical terms, and the need to translate into languages other than Chinese)?

☑yes ☐no

(3) Does the informed consent form require the signature of the subject or his or her legally authorized representative?

☑yes ☐no

(4) Is there any undue influence on the informed consent process?

☐yes ☑no

(5) Does the informed consent form contain mandatory language that compels the subject or the subject's representative to waive the legitimate rights and interests of the subject and absolves the investigator, sponsor, institution or its subordinates from liability for dereliction of duty?

☐yes ☑no

3. Whether the Information in the Informed Consent Is Consistent With the Description in the study Protocol and the Investigator's Brochure, Especially in the Following Aspects:

(1) Who should not participate in the study

☑yes ☐no

(2) Description of the study process, including trial procedures (particularly traumatic procedures), administration methods, doses, duration, etc

And medication regulations, etc

☑yes ☐no

propose：

☑agree

☐Agree after necessary corrections

☐Reexamine after necessary corrections
