# Supplementary material for: Association between the triglyceride-glucose index and hyperuricemia in patients with type 2 diabetes mellitus
Source: Front Endocrinol (Lausanne). 2025 Oct 17;16:1666563. doi: 10.3389/fendo.2025.1666563 (PMC12575192; doi:10.3389/fendo.2025.1666563)
Supplement: Supplementary file 2 [file Table1.doc]

Table S1. Steps for diagnosing collinearity

| Characteristics | All indicators | |
| --- | --- | --- |
|  | Tolerance | VIF |
| Age | 0.44 | 2.284 |
| Sex | 0.78 | 1.281 |
| Hypertension | 0.76 | 1.321 |
| BMI | 0.69 | 1.453 |
| SBP | 0.55 | 1.812 |
| DBP | 0.52 | 1.927 |
| HbA1c | 0.76 | 1.312 |
| TC | 0.86 | 1.169 |
| TG | 0.43 | 2.333 |
| HDL-C | 0.71 | 1.400 |
| LDL-C | 0.78 | 1.284 |
| Lp(a) | 0.90 | 1.108 |
| ALT | 0.29 | 3.416 |
| AST | 0.32 | 3.152 |
| FBG | 0.36 | 2.753 |
| UREA | 0.97 | 1.032 |
| Scr | 0.54 | 1.855 |
| eGFR | 0.37 | 2.716 |
| UACR | 0.96 | 1.045 |
| Ca | 0.73 | 1.379 |
| P | 0.78 | 1.275 |
| PTH | 0.84 | 1.193 |
| 25(OH)D | 0.87 | 1.153 |
| TyG.index | 0.21 | 4.665 |
