# Supplementary material for: Association between the triglyceride-glucose index and hyperuricemia in patients with type 2 diabetes mellitus
Source: Front Endocrinol (Lausanne). 2025 Oct 17;16:1666563. doi: 10.3389/fendo.2025.1666563 (PMC12575192; doi:10.3389/fendo.2025.1666563)
Supplement: Supplementary file 3 [file Table2.doc]

Table S2 Exclusion of the association between TyG and HUA in hypertensive population

| Variables | Unadjusted model | | Model 1 | |
| --- | --- | --- | --- | --- |
|  | OR (95%CI) | *P* | OR (95%CI) | *P* |
| TyG index | 1.32 (1.08 ~ 1.59) | 0.005 | 1.85 (1.07 ~ 3.20) | 0.027 |
| TyG index quantile |  |  |  |  |
| Q1 | 1.00 (Reference) |  | 1.00 (Reference) |  |
| Q2 | 1.36 (0.73 ~ 2.54) | 0.336 | 1.56 (0.69 ~ 3.50) | 0.286 |
| Q3 | 1.86 (1.02 ~ 3.37) | 0.042 | 2.69 (1.14 ~ 6.36) | 0.024 |
| Q4 | 2.55 (1.44 ~ 4.53) | 0.001 | 4.31 (1.48 ~ 12.52) | 0.007 |
| P for trend | 1.37 (1.14 ~ 1.63) | <.001 | 1.64 (1.17 ~ 2.31) | 0.004 |
| Model 1: Adjust: Age, Sex | | | | |
| Model 2: Adjust: Age, Sex, BMI, SBP, DBP, HbA1c, TC, TG, HDL.C, LDL.C, Lp.a, ALT, AST, FBG, UREA, Scr, UACR, Ca, P, PTH,25(OH)D | | | | |
| Model3: Adjust: Age, Sex, BMI, SBP, DBP, HbA1c, TC, TG, HDL.C, LDL.C, Lp.a, ALT, AST, FBG, UREA, Scr, UACR, Ca, P, PTH,25(OH)D，eGFR | | | | |
